# Functional two-way analysis of variance and bootstrap methods for neural synchrony analysis

**DOI:** 10.1186/1471-2202-15-96

**Published:** 2014-08-12

**Authors:** Aldana M González Montoro, Ricardo Cao, Nelson Espinosa, Javier Cudeiro, Jorge Mariño

**Affiliations:** Department of Mathematics, Facultad de Informática, Universidade da Coruña, Campus de Elviña s/n, 15071 A Coruña, Spain; Neuroscience and Motor Control Group (NEUROcom), Department of Medicine, Facultad de Ciencias de la Salud, Universidade da Coruña, Campus de Oza s/n, 15006 A Coruña, Spain

**Keywords:** Cross-correlation analysis, Bootstrap, Spike-trains, Dependence, Low firing-rate, Functional data

## Abstract

**Background:**

Pairwise association between neurons is a key feature in understanding neural coding. Statistical neuroscience provides tools to estimate and assess these associations. In the mammalian brain, activating ascending pathways arise from neuronal nuclei located at the brainstem and at the basal forebrain that regulate the transition between sleep and awake neuronal firing modes in extensive regions of the cerebral cortex, including the primary visual cortex, where neurons are known to be selective for the orientation of a given stimulus. In this paper, the estimation of neural synchrony as a function of time is studied in data obtained from anesthetized cats. A functional data analysis of variance model is proposed. Bootstrap statistical tests are introduced in this context; they are useful tools for the study of differences in synchrony strength regarding 1) transition between different states (anesthesia and awake), and 2) affinity given by orientation selectivity.

**Results:**

An analysis of variance model for functional data is proposed for neural synchrony curves, estimated with a cross-correlation based method. Dependence arising from the experimental setting needs to be accounted for. Bootstrap tests allow the identification of differences between experimental conditions (modes of activity) and between pairs of neurons formed by cells with different affinities given by their preferred orientations. In our test case, interactions between experimental conditions and preferred orientations are not statistically significant.

**Conclusions:**

The results reflect the effect of different experimental conditions, as well as the affinity regarding orientation selectivity in neural synchrony and, therefore, in neural coding. A cross-correlation based method is proposed that works well under low firing activity. Functional data statistical tools produce results that are useful in this context. Dependence is shown to be necessary to account for, and bootstrap tests are an appropriate method with which to do so.

## Background

The nervous system consists of a very large number of neurons—and glial cells—that are connected in complex networks. Neurons convey information by means of electrical pulses, called action potentials or spikes, consisting of a very fast and transient depolarization of their membrane potential. Sequences of spikes, called spike trains, are believed to serve as an information code in the brain. Pairwise synchrony between spike trains is widely studied in neuroscience as a way to understand these codes. Although neuronal interactions are probably not described only by pairwise associations [1], they have been shown to provide important information about neural coding [2–5]. On the other hand, the definition of neural synchrony is a matter of debate and different conceptions of it exist, such as “exact spiking coincidence” or “firing rate association”. There are several existing models for synchrony between simultaneous spike trains, most of them based on cross-correlation analysis. Common methods to measure synchrony are, for example, the cross-correlogram or the joint peristimulus time histogram [6]. Other methods not based on cross-correlation analysis include unitary event analysis [7, 8], conditional synchrony measure [9] and a method based on the distances between the closest spikes [10], among others. Most commonly, association measures are used to test for the presence/absence of synchrony. Several methodologies exist for this aim [11, 12]; however, in this paper we address a different problem. We use a cross-correlation based method, called the Integrated Cross-correlation Synchrony Index (ICCSI), which allows us to obtain a synchrony curve as a function of time and search for differences in the strength of pairwise associations regarding different factors. A similar problem was considered by Faes et al. [9], where these authors compare the synchrony between neurons under experimental conditions with synchrony at baseline.

During deep sleep, neurons in the cerebral cortex present a highly oscillatory global activity that can be observed by means of an electroencephalogram (EEG) or an electrocorticogram (ECoG). Furthermore, in the awake state, these global oscillations disappear giving rise to a less synchronized global activity. The transition from the sleep state to the awake state is regulated by the activating ascending pathways, which are afferent neuronal pathways originating in nuclei located at the basal forebrain and the brainstem [13, 14]. Under general anesthesia, the EEG is characterized by the presence of a number of patterns (spindles, K-complexes, delta waves) that show a progressive increase in low-frequency, high-amplitude activity [15–17]. In this scenario, the transition to the awake-like state can also be reproduced by means of microelectrical stimulation of some activating ascending areas [18]. On the other hand, an important property of neurons in the primary visual cortex (V1) is orientation selectivity; i.e., the neurons respond better (with a higher firing rate) to a specific orientation of the visual stimulus, the so-called preferred orientation [19]. This property is important in neurophysiological studies because it can provide meaningful clues regarding the physiology and microanatomy of the striate cortex.

The aim of the present study is to investigate the differences in synchrony strength regarding different experimental conditions given by anesthesia and awake-like activity, as well as the orientation selectivity of neurons in V1: we study whether similarities among neurons, such as the similarity in preferred orientation, affect the strength of correlated activity. We perform the analysis in a group of neurons recorded from V1 of an anesthetized adult cat. A method is proposed to investigate whether the mode of activity (anesthesia or awake-like), and the affinity in orientation selectivity of a given pair of neurons, have a determinant influence in how neuronal synchronization evolves.

The data are synchrony measurements computed at any time point. Therefore, the data are curves varying continuously over time. This is the reason why, in this paper, the word *functional* will be used to denote the nature of the data and not to make reference to the neurophysiology of the neurons under study. This term comes from statistics, where “functional data analysis” is a widely developing research field.

A functional two-way analysis of variance is proposed. Functional data analysis tools, based on Cuesta-Albertos and Febrero-Bande [20], are used. The method is adapted to consider the dependence that exists among the data because of the experimental setting. A parametric bootstrap is proposed for hypothesis testing.

## Methods

In this section, the data are presented. Also, the synchrony measure used to obtain the functional data is described, as well as the statistical methodology used to cope with the functional analysis of variance (ANOVA) model.

### Dataset

Data were recorded from an anesthetized and paralyzed adult cat. A microelectrode array with eight independent movable electrodes was introduced into the primary visual cortex of the animal for neuronal recording. Another two microelectrodes were introduced into the brainstem and basal forebrain for electrical stimulation. These stimulations, which we denote as *bs* (when the brainstem is stimulated) and *bf* (when the basal forebrain is stimulated), provoked a change in cortical activity from anesthesia to an awake-like pattern. All experiments followed the guidelines of the International Council for Laboratory Animal Science and the European Union (statute nr 86/809) and the protocols were approved by the University of A Coruña Committee on Animal Care.

At the beginning of each recording, neurons were characterized regarding their preferred orientation. Drifting gratings were used to visually stimulate the cat while the firing activities of a group of neurons were recorded. Each grating corresponded to an angle, which we call orientation, with a specific direction of movement. Orientation (and direction) are continuous variables; however, owing to the nature of the experiments, they will here be considered as discrete. Sixteen possible orientation-direction gratings were used: eight orientations with two possible directions each. For example: a drifting grating at 90° (the lines composing the grating are, therefore, vertical) that moves from right to left is a possible orientation-direction stimulus; another moving from left to right is a different one. Although the use of the two possible directions is also of interest in the study of other properties of V1 neurons (for example, the selectivity to direction), in this work we focus our analysis on the orientation selectivity. Hence, there were eight possible values for orientation: 0°,22°,45°,67.5°,90°,112.5°,135° and 157.5°. So, each recorded neuron was associated with one orientation (the preferred one), corresponding to its maximum firing rate. However, we still need to go one step further, as the objective of the study is to evaluate the effect of orientation selectivity on neural synchrony. To achieve this aim, each pair of neurons is identified with a value of a variable, *G*, which is defined as the difference between the preferred orientations of the neurons that form the pair. That is, if *O*_1_ and *O*_2_ are the preferred orientations of two neurons, we define *G*=min{|*O*_1_−*O*_2_|,180°−|*O*_1_−*O*_2_|}. Given the previous considerations, *G* can take one of these five possible outcomes: 0°,22.5°, 45°, 67.5° and 90°.

Throughout the paper, we will denote the number of neurons in a simultaneously recorded group by *n* and the number of possible pairs by . The number of experimental conditions will be denoted by *K*, the number of trials in each of these conditions will be *L* and *N* will denote the total sample size: *N*=*KrL*. For our data, *n*=8, *r*=28, *K*=2, *L*=4 and *N*=224. The firing rates of the test neurons varied from less than 1 Hz to around 7 Hz; however the most common values ranged from 1 to 3 Hz, denoting typically low firing activity.

### Synchrony measure

To measure synchrony, we used a cross-correlation based measure defined as the area under the cross-correlation function in a neighborhood around zero. Data were recorded under spontaneous activity, which is characterized by its very low firing activity. Exact spiking coincidences hardly ever occur, although the global activity of the brain is highly synchronized. By considering the area under the normalized cross-correlation function in an interval around zero we allow for a more relaxed definition of synchronous spike trains than that of just exactly coincident events.

#### Integrated cross-correlation synchrony index (ICCSI)

Let  and  be two simultaneously recorded spike trains in the time interval [0,*T*]. That is,  is the time when the *i*-th spike of train 1 occurred, and similarly for . Let *U*_*i*_ and *U*_−*i*_ be the *waiting times* from a spike in train _1_ to the *i*-th subsequent and the *i*-th preceding spike in train _2_, respectively. The probability density functions of these random variables are called the forward and backward cross-interval densities of order *i*, respectively, and we denote them by *η*_*i*_(*τ*) and *η*_−*i*_(*τ*). The cross-correlation function *ζ*(_1_,_2_;*τ*) is defined as the sum of cross-interval densities of all orders:
1

where *τ* is an arbitrary value, the waiting time. The normalized cross-correlation function


represents the probability density function of the time from an event in train _1_ to an event randomly chosen in train _2_ [21].

The observed spike trains can be used to estimate the cross-interval densities. Empirical normalized cross-correlograms are used to estimate the normalized cross-correlation function. The cross-correlogram, , is built as the histogram (or a smooth version, such as a kernel estimation) of the observed *waiting times* between the spikes of the first neuron and the spikes of the second neuron. Usually, joint firing, or close-in-time firing, is the event of interest so only small values of *τ* in (1) are taken into account. Then, we consider the normalized (in [−*v*,*v*]) cross-correlogram, , as follows:


The intuitive definition of synchrony involves the event of two neurons firing together. Under low firing rates, the spikes corresponding to two highly synchronized neurons do not appear exactly at the same time, although they may follow a similar pattern. In this context, flexible tools are needed to capture synchrony. We use the integral of the cross-correlogram around zero:
2

where *δ*<1 is an arbitrary small number chosen by the researcher. In this way, we allow synchrony to be based on delayed firing and not only on simultaneous firing. Integrating  in a neighborhood of zero we account for spikes that occur closely in time, though not exactly at the same time.

To study the evolution of synchrony in time, sliding windows can be used. That is, if the total observational time of the spike trains is [0,*T*], synchrony at time *t*∈[*w*,*T*−*w*] can be estimated by computing *Y*(_1_,_2_) in the time window (*t*−*w*,*t*+*w*]. Using a time grid, 0<*t*_1_<…<*t*_*M*_<*T*, and computing the ICCSI at each time point of the grid, synchrony becomes a function of time: *Y*(_1_,_2_;*t*). Details on functional data analysis are not given here, though we outline the basic notions that are necessary to understand our analysis. For details and theory on this subject, we refer the reader to, for example, the books [22–24].

**Figure 1:**
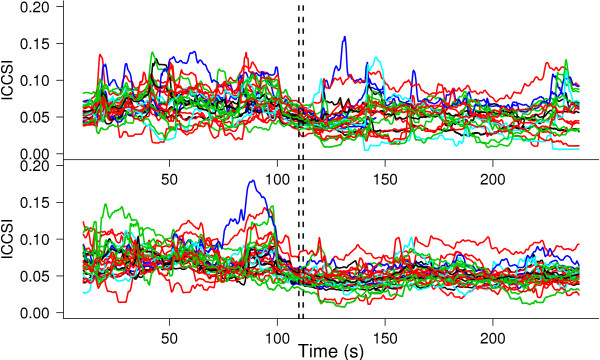
**Functional data.** Top panel: ICCSI curves (synchrony between pair of neurons along time) averaged over four trials for the first stimulus, *bs*. Bottom panel: ICCSI curves averaged over four trials for the second stimulus, *bf*. Different orientation selectivity groups are shown in different colors: 0° (black), 22.5° (red), 45° (green), 67.5° (blue) and 90° (cyan).

The number of pairs in each of the categories given by *G* (0°, 22.5°, 45°, 67.5° and 90°) is 5,12,6,3 and 2 respectively. Therefore, there are 20,48,24,12 and 8 curves in each category defined by stimulus and orientation. The curves can be evaluated over as many points as desired. Points in an equispaced grid 0<*t*_1_<…<*t*_*M*_=*T* are considered: from 10 s to 230 s every 0.1 s. Therefore, each synchrony curve is evaluated over 2201 points. Let *Ψ*={(*i*,*j*):*i*,*j*∈1,…,*n* and *i*<*j*} and denote the pairs of neurons with indices (*i*,*j*)∈*Ψ*. We will denote by  the *l*-th trial for the curve *Y*(_*i*_,_*j*_;*t*) under the *k*-th stimulus; *k*=1,2 and *l*=1,2,3,4. The curves are bounded, since . Figure 1 shows the data averaged over trials. The top panel shows the functions that correspond to *bs* stimulation and the bottom panel shows the ones for *bf* stimulation. Different colors are used for the different levels of *G*.

We search for population differences in the dynamics of the awake-like period induced by each stimulus, taking into account the possible effect of the other factor: difference between orientation selectivity. This problem can be dealt with as a functional two-way ANOVA with a two-level factor: stimulus and a five-level factor: *G*.

### Functional ANOVA

As already mentioned, the aim of this work is to search for differences in synchrony dynamics relative to two factors: stimulus and difference in orientation selectivity. As the difference in orientation selectivity can take only values in a given finite set of values, the problem is one of a two-way analysis of variance with fixed effects in which the response variable is functional. In the following subsection, we outline the methods.

#### The random projection method for the ANOVA model

The random projection approach in the functional data context is based on the ideas of Cuesta-Albertos et al. (2007) [25]. These authors give an extension, on Hilbert spaces, to the Cramer-Wold theorem, which characterizes a probability distribution in terms of one-dimensional projections. Their Theorem 4.1 states that if two distributions are different, and we choose a marginal of them at random, those marginals will almost surely be different. Based on this fact, Cuesta-Albertos and Febrero-Bande [20] propose a method for hypothesis testing in infinite dimensional spaces. We will state their result more formally, particularizing it to our problem. Let us assume the data belong to a Hilbert space,, with a scalar product 〈, 〉, and let *μ*_*G*_ be a Gaussian distribution in. Suppose the hypothesis to be tested is whether certain parameters, say *γ*_1_ and *γ*_2_, are equal (*H*_0_:*γ*_1_=*γ*_2_). If *γ*_1_≠*γ*_2_, then the set of random directions, *ν*, from *μ*_*G*_ in, for which 〈*ν*,*γ*_1_〉=〈*ν*,*γ*_2_〉, has probability zero. That is, if *H*_0_ fails, then it also fails in its projected version for *μ*_*G*_—almost every *ν*∈. Therefore, a test at level *a* in the one-dimensional space is a test at the same level to test *H*_0_.

We now present the functional two-way ANOVA model for our problem and state the methodology more formally. We consider the following linear model for the synchrony curves:
3

for *k*=1,2 and *l*=1,2,3,4. The function  is the ICCSI for trial *l* of the pair given by neurons *i* and *j* under stimulus *k*; *m*(*t*) is the global effect curve and *α*_*k*_(*t*) is the effect of stimulus *k*. The function *g*:*Ψ*↦{1,2,⋯,5} indicates the level of the factor *G* that corresponds to the pair given by neurons *i* and *j*, identifying level 1 to 0°, level 2 to 22.5° and so on. Therefore, *β*_*g*(*i*,*j*)_ is the effect of level *g*(*i*,*j*) on the synchrony curve. The effect of a possible interaction between the factors is gathered by *γ*_*kg*(*i*,*j*)_ and, finally,  is the random error term. For parameter identifiability, we assume:
4

The relevant null hypotheses to be tested are:


which means that there is no effect of the stimulus and


which states that there is no effect of the orientation selectivity. Also, a hypothesis for the interactions is interesting:


Theorem 2.1 in [20] states that, if the data belong to a Hilbert space,, endowed with a scalar product 〈, 〉, *μ*_*G*_ is a Gaussian distribution on such that its one-dimensional projections are non-degenerate, then,

1. If ∃*k*∈{1,2}, such that *α*_*k*_≠0, then
2. If ∃*g*∈{1,2,3,4,5}, such that *β*_*g*_≠0, then
3. If ∃*k*∈{1,2} and *g*∈{1,2,3,4,5} such that *γ*_*kg*_≠0, then

Therefore, the proposed procedure is to randomly project the data on the one-dimensional space and to test the hypotheses in that context. Given a random function, *ν*(*t*), and denoting the projection of a function *f*∈ in the direction of *ν* as *f*^(*ν*)^, 〈*ν*,*f*〉, we consider the projected model:
5

and the hypotheses in the one-dimensional problem:
6

The tests defined in the one-dimensional response case are clearly conditional on the random projection used, *ν*. To reduce the error introduced by the choice of the random projection, we will use the correction that implies controlling the false discovery rate (FDR) introduced by Benjamini and Hochberg (1995) [26]. This method is also recommended by Cuesta-Albertos and Febrero-Bande [20]. In particular, we use the FDR procedure that arises from the work of Benjamini and Yakutieli (2001) [27]. Given the ordered p-values *p*_(1)_<…<*p*_(*s*)_ obtained using *s* random projections, we will choose the corrected p-value as the quantity , where min stands for the minimum of a set.

So far, this test can help in the search for global differences between the two groups of curves, although we would rather study how these differences change in time. To do this, we propose the use of moving windows along time. For each time point, *t*, we consider an interval of time, centered at *t*, and project the pieces of curves that correspond to that interval, to perform the ANOVA test.

The hypotheses in (6) can be tested with any regular ANOVA approach. Nevertheless, some caution is needed, as the errors in our model cannot be assumed to be independent, as we discuss in the next subsection.

#### ANOVA model with dependent errors

In this section we introduce the problem of the dependence that is present in the data. The dependency comes from the fact that the data are observed at the neuron-pair level. So, it is only fair to think that the curves obtained from two pairs of neurons with one cell in common could be correlated.

Since we work with the projections of the *Y* functions, we can forget about the infinite dimensional problem and focus on the one-dimensional one. Let us consider the model in (5), dropping the superscript (*ν*) for simplicity:
7

with (*i*,*j*)∈*Ψ*, *k*∈{1,2} and *g*∈{1,2,…,5}. Model (7) is a two-way ANOVA model with a two-level first factor and a five-level second factor, with unbalanced cells, as we do not observe the same amount of pairs of neurons with differences in orientation selectivity. The following representation of the problem is more convenient. Consider each , i.e., the synchrony of a given pair of neurons under each stimulus, as a random variable. In this way, we have four realizations of each variable, so our linear model becomes another linear model with 2*r* variables and *L* observations each. The model can be written in matrix form, as follows:
8

where  are the data ordered in a convenient form,  is the design matrix,  is the vector of parameters, *θ*^*T*^=(*m*,*α*_1_,*β*_1_,*β*_2_,*β*_3_,*β*_4_,*γ*_11_,*γ*_12_,*γ*_13_,*γ*_14_,*γ*_15_,*γ*_21_,*γ*_22_,*γ*_23_,*γ*_24_). The parameters *α*_2_, *β*_5_ and *γ*_25_ are not included in the definition of *θ* because they are superfluous by the conditions defined in (4). Finally,  is the vector of errors. Here, we consider the data ordered as follows:


and therefore the vector of errors, **ε**, has the form:


The assumptions of normality and homoscedasticity for the errors are reasonable in this context, but the fundamental problem in this study arises from the presence of dependence among the data that comes from pairs of neurons sharing a cell. That is,  and  are dependent if {*i*,*j*}∩{*i*^′^,*j*^′^}≠*∅*. The errors of the model are normally distributed with zero mean and covariance matrix **Σ**. We assume that the variance of  is the same for all (*i*,*j*) and all *k* and equal to *σ*^2^. On the other hand, we also assume that  whenever *#*({*i*,*j*}∩{*i*^′^,*j*^′^})=1, where *#* denotes the cardinal (number of elements of a set). Let *Ω*={(*i*,*j*,*k*,*l*,*i*^′^,*j*^′^,*k*^′^,*l*^′^):*#*({*i*,*j*}∩{*i*^′^,*j*^′^})=1,*k*=*k*^′^,*l*=*l*^′^} then, in summary:
9

Therefore, **Σ** results in a very special matrix, with *σ*^2^ in the diagonal, and *σ*^2^*ρ* where the variable in the column and the variable in the row share a neuron and also share a trial (and, thus, a stimulus). That is, **Σ** is a *N*×*N* matrix composed by a diagonal of *nr*×*r* blocks, and the rest of the elements are zeros. The blocks in the diagonal are all equal and equal to the covariance matrix, *σ*^2^**C**, of the data that correspond to one level of the first factor (stimulus):
10

With *C*=*C*(*ρ*) in our particular example (*r*=28):


With this definition, **Σ** is positive definite for small values of *ρ*. The eigenvalues of the matrix in (10) result in only three different values: (1−2*ρ*)*σ*^2^, (1+4*ρ*)*σ*^2^ and (1+12*ρ*)*σ*^2^, which, for *ρ*∈[0,1], are all greater than zero at the same time, whenever *ρ*<0.5. This means that this correlation structure for the data is plausible only for *ρ*∈ [ 0,0.5).

#### The *F*statistic

Our ANOVA problem comprises the hypothesis test that some of the parameters in model (8) are equal to zero. In matrix form, this can be written as
11

where, in the case of the stimulus effect,


and, in the case of the orientation selectivity, the hypothesis matrix is


and, in both cases, **h** is a vector of zeros. Let us denote by *q* the total number of parameters in the complete model and let *q*_0_ be the number of parameters, different from zero, under the null hypothesis.

Let the residual sum of squares function, in matrix form, be denoted by **Q**(*θ*):


Denote by  the estimate of *θ* in the complete one-dimensional model and let  be the estimate for the reduced model. That is,  is the vector of parameters that minimizes **Q(θ)** under the restriction given by (11). Then, the classical ANOVA **F** statistic is
12

where *d*_1_ denotes the degrees of freedom of the numerator, that is, *q*−*q*_0_, and *d*_2_ the degrees of freedom of the denominator: *N*−(*q*+1).

The test statistic, **F**, under assumptions of independence, normality and homoscedasticity, follows an *F* distribution with *q*−*q*_0_ and *N*−(*q*+1) degrees of freedom. In our context, as the errors of the model cannot be assumed to be independent, the test statistic does not have an  distribution and, therefore, we need to calibrate the null distribution of the test statistic. We propose a parametric bootstrap procedure to calibrate the distribution of the ANOVA test statistic under the null hypothesis.

#### Estimation of the correlation coefficient

Since we assume that the covariance between the different pairs of error terms are equal, provided the pairs belong to *Ω*, we will estimate the correlation coefficient as the average of the Pearson correlation coefficient of the corresponding pairs, which is equivalent to:
13

where  are the elements of the residual vector:  and  is the estimated residual variance:


#### Direct bootstrap

As the data are normally distributed, we propose the use of a parametric bootstrap to calibrate the distribution of the ANOVA test statistic under the null hypothesis. We now describe the procedure for a general null hypothesis .

Once the linear model has been fitted to obtain  and the classical ANOVA statistic has been computed (denoted by **F**^*obs*^), we estimate *σ*^2^ and *ρ* from the residuals to build the estimated covariance matrix, . We proceed with the following bootstrap algorithm:

1. Replace the *i*-th parameter in the estimated  by zero (null hypothesis). This set of parameters will be denoted by . Build a bootstrap sample:  with .
2. Fit the linear model to the resample, obtain the bootstrap version of the estimated parameters  and compute **F**^∗^, the bootstrap version of **F**.
3. Repeat Steps 1–2 *B* times to obtain **F**^∗1^,…,**F**^∗*B*^.
4. Compute the desired (1−*a*)-quantile of the bootstrap versions, . We reject the null hypothesis if .

## Results and discussion

In this section we show the results of applying the random projections method to the functional ANOVA problem at hand. To draw the random vectors we use Brownian motions or, more precisely, approximations to standard Brownian motions by sequences of partial sums of normally distributed random variables. We only need to compute the values of the random vectors in the equidistant time points, *t*_1_,⋯,*t*_*M*_, where the functions  are defined. For this, we consider *M* independent and identically distributed standard normal random variables, *Z*_1_,…,*Z*_*M*_, and define a trajectory *ν*_1_ as
14

On the other hand, we would like to have directions without tendency and such that their variability throughout the trajectory does not change too much. For this aim, we define the random trajectories as the sum of two Brownian motions, as just defined, where one of them has been “flipped" so as to be equal to zero in the last time point *t*_*M*_. That is, let *ν*_1_ and *ν*_2_ be defined as in (14) and let *ν*_3_(*t*_*k*_)=*ν*_2_(*t*_*M*−*k*+1_). The final directions we use are defined as *ν*(*t*)=*ν*_1_(*t*)+*ν*_3_(*t*).

**Figure 2:**
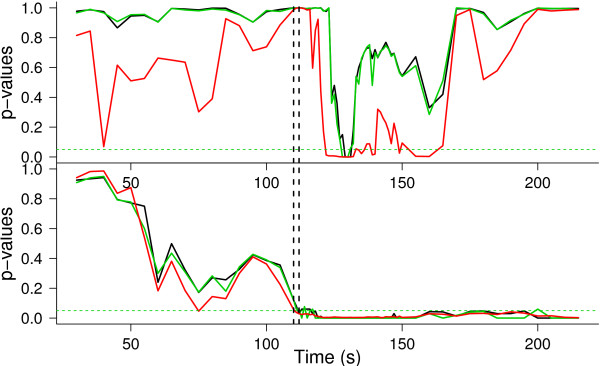
**ANOVA** ***p*** **-values.** *p*-values for the two-way ANOVA as a function of time, for the significance of the stimulus effect (top panel) and for the significance of the difference in orientation selectivity (bottom panel). *p*-values obtained using the  distribution (red), direct bootstrap (black) and *χ* ^2^-based bootstrap from the appendix (green) are shown. *p*-values below the horizontal dotted line (constant value 0.05) are significant. The vertical dotted lines depict the stimulation times.

A preliminary analysis, fitting model (7), showed that the interaction between factors was not significant. Therefore, the final model considered is:
15

with *k*∈{1,2}, *g*(*i*,*j*)∈{1,2,…,5}, and *l*∈{1,2,3,4}. Figure 2 shows the p-values obtained by using sliding windows across time to study the evolution of the effects of both factors. A 40-s time window was considered, moving along the time axis (in seconds) from 20 s of recording to 215 s. In the time period between 110 s and 150 s, this was done every second; for the rest, it was done every 5 s. At each window, 30 random directions were used to project the data (the same ones in every window) and the FDR correction was applied, resulting in just one p-value. It is clear that there are differences between the two approaches used. When dependence is not taken into account (red lines) the test is less conservative than when dependence is included. Although for the effect of the stimuli there is a period of time at the beginning of the awake-like period (right after stimulation) for which both tests reject the null hypothesis, next there is another period in which it would be rejected if dependence was not accounted for. The results show that a period of time exists during the awake-like mode when the difference between the effects of the two stimuli is significant. This result reinforces the view that there are important differences in the physiology and dynamics of the two activating pathways: *bs* and *bf*. On the other hand, the differences in synchrony among the levels of the factor *G* were also found to be significant after the stimulus.

**Figure 3:**
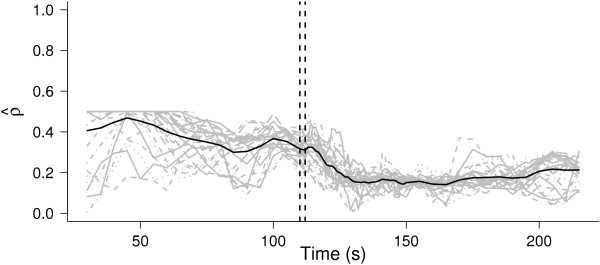
**Correlation coefficient estimates.** Evolution of the correlation coefficient estimates. Estimations for different random projections (grey lines), and their means (black line). The vertical dotted lines depict the stimulation times.

The estimation of the correlation coefficient changes for each window; nevertheless, the estimation is not very variable, even from one projection to the other. Figure 3 shows the  as a function of time and their mean across projections. We can observe that, at the beginning of the recording, the estimated correlation coefficients were greater than 0.5 and they were truncated for the covariance matrix, , to be positive definite.

When large correlation is present (*ρ*>0.5) an alternative, nonparametric, bootstrap can be carried out. In the following procedure, each set of bootstrap residuals is defined as the set of original residuals of a trial chosen at random with equal probability from all possible trials:

1. For each *k*∈{1,2} and *l*∈{1,2,3,4} draw the bootstrap pair (*k*^∗^,*l*^∗^) with equal probability from {1,2}×{1,2,3,4}, that is,  for all *k*^′^∈{1,2,} and all *k*^′^∈{1,2,3,4}.
2. Define .

This bootstrap procedure has the drawback that, in our case, the vector of bootstrap residuals can only take eight possible values. A possible improvement is to use a smoothed version. To achieve this, a smoothing parameter *h*, typically small with respect to the standard deviation of the residuals, is chosen and Step 2 is replaced by:

2. Define  with  iid ∀(*i*,*j*).

The green curve in Figure 2 represents the *p*-values obtained with an alternative bootstrap algorithm based on an approximation to the real distribution of the *F* statistic. This alternative method, which we call the *χ*^2^-based bootstrap, reduces computational time considerably. It involves some theoretical insight that we describe in the Appendix. The main result is based on the fact that the test statistic **F** can be expressed as a ratio of quadratic forms on the errors of the model. There are plenty of results concerning the exact distribution of quadratic forms on normal vectors. For example, Provost et al. [28] derive the exact density function of an indefinite quadratic form in noncentral vectors, which allows us to derive the distribution of ratios of quadratic forms as those involved in ANOVA tests. Nevertheless, the closed formulas for the density functions of quadratic forms are complex and not practical. On the other hand, the distribution of the numerator and the denominator that give shape to the **F** statistic are quite easy to approximate by Montecarlo, as is also described in the Appendix. We use this approach to carry out, in the next two sections, an evaluation of the test. First, we compare the distribution of the test statistic when dependence is either taken into account or not. Finally, we perform a simulation study to evaluate the performance of the bootstrap test.

### Distribution of the test statistic **F**

**Figure 4:**
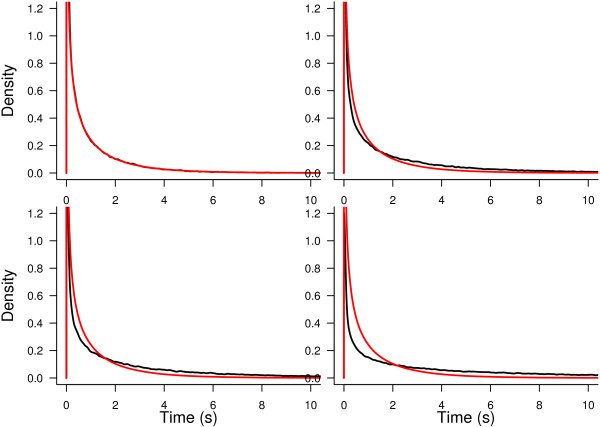
**Test statistic density function under no stimulus effect.** Probability density function of the test statistic under no stimulus effect (black lines) compared with the corresponding  distribution (red lines) under different correlation scenarios: *ρ*=0 (top-left panel), *ρ*=0.1 (top-right panel), *ρ*=0.15 (bottom-left panel) and *ρ*=0.4 (bottom-right panel).

**Figure 5:**
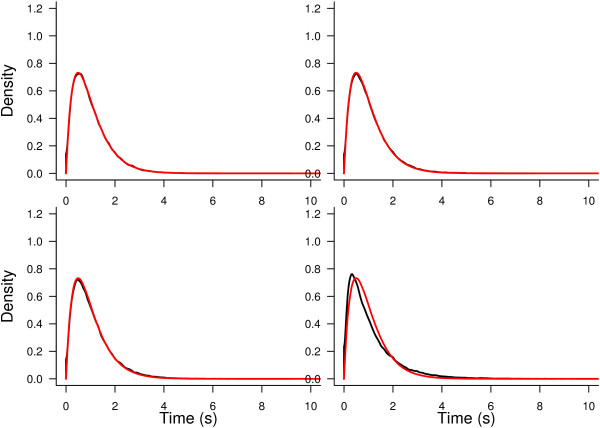
**Test statistic density function under no effect of the difference in orientation selectivity.** Probability density function of the test statistic under no effect of the difference in orientation selectivity (black lines) compared with the corresponding  distribution (red lines) under different correlation scenarios: *ρ*=0 (top-left panel), *ρ*=0.1 (top-right panel), *ρ*=0.15 (bottom-left panel) and *ρ*=0.4 (bottom-right panel).

To visualize the differences between the distribution of the test statistic, **F**, either when taking into account dependence or when independence between observations is assumed (*F* distribution), Figures 4 and 5 show the density of **F** under the null hypotheses, approximated by Montecarlo in the numerator and denominator of (20). The model used is the same as in the real data case; i.e., **y**=**X***θ*+*ε* given by (15). The real data scenario was reproduced by constructing the model for eight neurons and four trials under each stimulus. The values of the second factor were exactly as in the real case. Moreover, the errors were assumed *ε*∼*N*(0,**Σ**) with **Σ** defined as in (10) with *σ*^2^=1 and different values for the correlation coefficient: *ρ*=0,0.1,0.15 and 0.4. The first panel of Figures 4 and 5 show that the null distribution of the test statistic corresponds to the  distribution (as it should) when *ρ*=0. Figure 4 shows that, on the other hand, the  distribution departs from the null distribution of the test statistic when *ρ* increases. This is evidence for the necessity of using the bootstrap to calibrate the distribution of **F** instead of using the *F* distribution. Figure 5 shows the comparison of both densities for the hypothesis on *β*_*i*_. In this case the difference is not as large as in the previous case. However, in the last panel we can still observe a small deviation. In general we can state that, when *ρ* increases, the tail of the distribution becomes heavier. This explains why, sometimes, the test using the classical approach rejects while the bootstrap approach does not.

### Performance of the test

In this section we perform a simulation study to evaluate whether the type I error (probability of rejecting the null hypothesis given that it is true) of our test is close to its nominal value (*a*=0.05). Also, we evaluate the power of the test, which is the probability that it correctly rejects the null hypothesis when the null hypothesis is false. For this aim, we simulated data, similar to real data, using different (known) model parameters and correlation coefficients. With the simulated data, we computed **F** and followed the procedure used to calibrate the distribution of the test statistic with level *a*=0.05. Finally, we compared the results (acceptance/rejection of the null hypothesis) with the true case. The data were generated as if three trials under two stimulation conditions of a group of seven neurons had been recorded. Each neuron was assigned a given fixed characteristic (orientation selectivity) so that the second factor (difference in orientation selectivity) could be computed. In this case, the preferred orientations were defined with only two possible values that were assigned arbitrarily to each neuron. Then,


where **θ**=(*m*,*α*_1_,*β*_1_)^*t*^. The covariance matrix, **Σ**, was defined as in (9) for four values of *ρ*. The values used for the simulations are *m*=0, *α*_1_=0,0.25,0.5, *β*_1_=0,0.25,0.5 and *ρ*=0,0.05,0.15,0.35.

A large variance (*σ*^2^=1) with respect to the parameters was chosen to reflect a typical situation for the real data. For most of the projections, the signal to noise ratio is rather small. This large variance might affect the ability of the test to detect differences between the parameters. *M*=5000 Montecarlo simulations were performed and for each one, *B*=500 bootstrap trials were used. The results are shown in Tables 1, 2 and 3. Table 1 shows the proportion of rejections when testing for no effect of the first factor. It can be observed that the nominal level of the test is not well respected under dependence. The test is moderately anti-conservative when using the bootstrap, and severely anti-conservative when using the *F* distribution. When using the bootstrap, the rejection percentage under the null remains close to the nominal even when increasing the dependence. This is not the case when using the *F* distribution, since the rejection percentage under the null is inadmissibly large. Table 2 shows the proportion of rejections when testing for no effect of the second factor. In this case, the bootstrap respects the nominal level reasonably well when testing for *β*_1_. However, the test based on the *F* distribution is very conservative under large dependence. Regarding the power of the test, Table 1 shows that the proportion of rejections decreases when *ρ* increases for both tests. It is noticeable that the power of the test based on the *F* distribution is rather larger than that of the bootstrap test, especially for large correlations. Nevertheless, the fact that the level of the test is respected a lot better by the bootstrap makes this method more appropriate. Table 2 shows that, surprisingly, the value of the correlation parameter does not influence the results, regarding power, when testing for *β*_1_. For *β*_1_=0.5, we can observe near to 100*%* rejection under the alternative in almost all cases. Table 2 also shows how the power of the bootstrap test is better compared with the one calibrated with the *F* distribution.

**Table 1:** **Proportion of rejections for**  **at level** ***a*** **=0** ***.*** **05**

		***ρ***=0		***ρ***=0 ***.***05		***ρ***=0 ***.***15		***ρ***=0 ***.***35	
		Boot.	***F***	Boot.	***F***	Boot.	***F***	Boot.	***F***
	*β*_1_=0	0.0456	0.0512	0.0804	0.1156	0.0828	0.2166	0.0816	0.3692
*α*_1_=0	*β*_1_=0.25	0.0378	0.0464	0.0764	0.1102	0.0874	0.2258	0.0806	0.3680
	*β*_1_=0.5	0.0464	0.0536	0.0756	0.1092	0.0862	0.2178	0.0848	0.3780
	*β*_1_=0	0.7570	0.7906	0.6482	0.7476	0.4864	0.7044	0.3164	0.6762
*α*_1_=0.25	*β*_1_=0.25	0.7564	0.7920	0.6590	0.7598	0.5022	0.7124	0.3204	0.6644
	*β*_1_=0.5	0.7548	0.7886	0.6342	0.7390	0.4860	0.7050	0.3164	0.6810
	*β*_1_=0	0.9996	0.9998	0.9940	0.9982	0.9430	0.9882	0.7794	0.9602
*α*_1_=0.5	*β*_1_=0.25	0.9996	1.0000	0.9944	0.9988	0.9478	0.9970	0.7858	0.9596
	*β*_1_=0.5	0.9998	0.9998	0.9950	0.9986	0.9460	0.9898	0.7666	0.9568

**Table 2:** **Proportion of rejections for**  **at level** ***a*** **=0** ***.*** **05**

		***ρ***=0		***ρ***=0 ***.***05		***ρ***=0 ***.***15		***ρ***=0 ***.***35	
		Boot.	***F***	Boot.	***F***	Boot.	***F***	Boot.	***F***
	*α*_1_=0	0.0588	0.0532	0.0558	0.0462	0.0444	0.0238	0.0376	0.0032
*β*_1_=0	*α*_1_=0.25	0.0566	0.0538	0.0512	0.0448	0.0462	0.0244	0.0414	0.0032
	*α*_1_=0.5	0.0516	0.0460	0.0484	0.0422	0.0448	0.0248	0.0374	0.0040
	*α*_1_=0	0.7936	0.7844	0.8094	0.7926	0.8838	0.8358	0.9758	0.8858
*β*_1_=0.25	*α*_1_=0.25	0.7938	0.7864	0.8280	0.8072	0.8716	0.8152	0.9796	0.9020
	*α*_1_=0.5	0.7830	0.7740	0.8118	0.7940	0.8824	0.8298	0.9802	0.9038
	*α*_1_=0	1.0000	1.0000	1.0000	1.0000	1.0000	0.9998	1.0000	1.0000
*β*_1_=0.5	*α*_1_=0.25	0.9998	.9998	0.9998	0.9996	1.0000	1.0000	1.0000	1.0000
	*α*_1_=0.5	1.0000	1.0000	0.9998	0.9996	1.0000	1.0000	1.0000	1.0000

**Table 3:** **Proportion of rejections for**  **at level** ***a*** **=0** ***.*** **05**

		***ρ***=0		***ρ***=0 ***.***05		***ρ***=0 ***.***15		***ρ***=0 ***.***35	
		Boot.	***F***	Boot.	***F***	Boot.	***F***	Boot.	***F***
	*β*_1_=0	0.0558	0.532	0.0554	0.452	0.0454	0.0254	0.0382	0.0036
*α*_1_=0	*β*_1_=0.25	0.0568	0.532	0.0506	0.426	0.0458	0.0248	0.0386	0.0052
	*β*_1_=0.5	0.0500	0.474	0.0476	0.394	0.0478	0.0264	0.0410	0.0038
	*β*_1_=0	0.0610	0.0552	0.0510	0.0414	0.0482	0.0274	0.0396	0.0032
*α*_1_=0.25	*β*_1_=0.25	0.0530	0.0500	0.0494	0.0402	0.0442	0.0260	0.0334	0.0036
	*β*_1_=0.5	0.0502	0.0500	0.0496	0.0414	0.0472	0.0280	0.0374	0.0028
	*β*_1_=0	0.0516	0.0464	0.0476	0.0408	0.0424	0.0236	0.0330	0.0024
*α*_1_=0.5	*β*_1_=0.25	0.0582	0.0540	0.0516	0.0430	0.0440	0.0242	0.0414	0.0056
	*β*_1_=0.5	0.0524	0.0480	0.0552	0.0438	0.0500	0.0298	0.0370	0.0052

Finally, we show similar simulation results when testing for the interaction between the two factors. Table 3 shows the proportion of rejections under the null hypothesis for different values of *ρ* and 5000 Montecarlo replications. We can observe that the nominal level is successfully met by the bootstrap test for small correlation values. Although for the case of large correlation values the test seems to be conservative, it outperforms the *F* test remarkably. It is important to note that, regarding the power of the test, in these simulations the test rejected the null hypothesis 100*%* of the time (results not shown), using values of *γ*=0.5,1 to simulate data in the alternative.

## Conclusions

In this work we proposed a functional two-way ANOVA for neural synchrony curves, using random projection techniques. These methods are very easy to implement and interpret, which makes them appealing for applying to many problems. The method was shown to be useful as it allowed the significance effects of the factors under study to be addressed.

The model under study involves synchrony curves obtained by a cross-correlation based method. The curves were separated into groups given by the stimuli and the difference in preferred orientation between the two neurons involved in each curve. Differences between the levels of this second factor were also of interest. Although there were groups with very few elements, several conclusions can be established.

The importance of including the dependence between curves in the analysis was shown. The distribution of the test statistic was approximated using a parametric bootstrap on the residuals of the model, allowing for dependence. The classical **F** test statistic can lead to false positives, as the distribution of the test statistic has a heavier tail than the *F* distribution. Two algorithms were presented to carry out the bootstrap: a direct resampling plan, which resamples from the model, and a second one, based on the fact that the **F** statistic has the same distribution as that of a ratio of particular linear combinations of  variables. The second algorithm has a substantially lower computational cost than the first one.

Regarding the real data, the interactions between the factors were not statistically significant. An effect of the factor *stimulus* can be found at the beginning of the awake-like period (a few seconds after the stimulus onset). To make a statement regarding the differential effect that *bs* and *bf* have on pairwise synchrony, the analysis of more groups of neurons would be necessary. However, this work shows that the question is worth asking, as we were able to find evidence of these differences after the switch from anesthesia to the awake-like mode. In fact, taking into account the ability of *bs* and *bf* to promote wakefulness with a similar effect on ECoG power spectral density [29], the robustness of the proposed statistical method (to be applied in low-activity cortical single units recorded simultaneously) allows us to find some differences in neural pattern synchrony, consistent with physiological data as follows. Activation of the parabrachial nucleus in the *bs* enables thalamic relay neurons to disrupt cortical synchronization via glutamate release [30]. In contrast, *bf* stimulation induces not only ACh release, but also GABA, glutamate and NO in the cortex [18, 31]. Thus, although the effect that *bs* and *bf* activation has on ECoG dynamics has been characterized as regulatory on cortical activation, the different operational mechanism of each system could be reflected in the temporal coordination between neurons. Finally, a relevant issue in neurophysiology relates to the type of information processing carried on by primary visual cortex neurons [32–34]. Briefly, some basic properties of the visual processing—like orientation selectivity—could be based either on highly refined and specific anatomical connections or, in contrast, could be carried out by distributed computational processes at the cortical level. The results presented here show that the effect of the difference in orientation selectivity was significant throughout all the generated awake-like activity, suggesting that the strength of the connectivity was dependent on the orientation selectivity of primary visual cortex neurons, thus favoring the second hypothesis.

Overall, this work is a contribution to the development of statistical tools for neuroscience. Although the methods we propose here have been focused on a particular problem, they are applicable in many other contexts. It is worth to mention that differences were found even though the firing activity of the test group was considerably low, showing the method can be useful when low firing rates are present. Also, bootstrap techniques are very powerful and easy to implement (although, admittedly, they can be computationally expensive), and might be a good alternative to parametric inference using minimal mathematical assumptions.

## Appendix - *χ*^2^-based bootstrap

Here we present the results that provide a computationally more efficient way to perform the bootstrap test. First of all, let us rewrite **F** in a more convenient form. Recall


where  is the estimator of the parameter vector *θ* in the unconstrained model, while  is the estimator under the null hypothesis (**H***θ*=**h**). It can be shown that  and  relate as follows:


where


and,


which implies that


Moreover, we can write this expression as a quadratic form based on the errors of the model by noticing that:


Therefore,


and finally,
16

In a similar way, the sum of squares of the denominator can be expressed as
17

From (16) and (17) we obtain:
18

Let us denote the matrix in the last expression of (18) by


and the final matrix in (17) by


Finally, we can give the expression for the **F** statistic in terms of quadratic forms on normal vectors:
19

where *ε*∼*N*(**0**,**Σ**).

Let us denote **Z**=*ε*^*t*^*A*_1_*ε*. Also, let **S** be a matrix such that **Σ**=**SS**^*t*^ and let **R**=**S**^−1^*ε*. We denote the eigenvalues of **S**^*t*^*A*_1_**S**, or equivalently those of **Σ***A*_1_, by *λ*_1_,…,*λ*_*N*_ and by **P**, an orthogonal matrix whose columns are the eigenvectors of **S**^*t*^*A*_1_**S**. So, **Z**=**R**^*t*^**S**^*t*^*A*_1_**SR**=**R**^*t*^**PDP**^*t*^**R**=*ε*^*t*^(**S**^−1^)^*t*^**PDP**^*t*^**S**^−1^*ε*=**V**^*t*^**DV**, where **V**=**P**^*t*^**S**^−1^*ε* and **D** is a diagonal matrix with diagonal elements *λ*_1_,…,*λ*_*N*_. It follows that **V**∼*N*(**0**,**I**) and, therefore,


where  for *i*=1,…,*N*.

The same argument is true for the quadratic form in the denominator of **F**. Thus, the distribution of *ε*^*t*^*A*_2_*ε* is the same as the distribution of , where *μ*_1_,…,*μ*_*N*_ are the eigenvalues of **Σ***A*_2_ and , *i*=1,…,*N*. Consequently, the ratio
20

has the same distribution as **F**. The distribution of (20) can be approximated by Montecarlo.

In practice, **Σ** is not known, so the *λ*_*i*_ and *μ*_*i*_ cannot be computed. However, **Σ** can be replaced by  as in (10). Thus,  and  (the eigenvalues of  and ) can be used in (20). Observe that this is equivalent to the original bootstrap resampling plan as presented above. This gives an alternative method for performing the bootstrap that is much less time consuming. For instance, in the example shown in Figure 2, the results obtained with the direct bootstrap are reproduced, except for minor differences due to Montecarlo error, and the computational time is at least 30 times smaller with this alternative method.
